# Dynamic change of IDO1 activity predicts survival in patients with unresectable stage III NSCLC and chemoradiotherapy

**DOI:** 10.3389/fimmu.2022.906815

**Published:** 2022-08-10

**Authors:** Linfang Wu, Daquan Wang, Yanhua Chen, Mingmin Qian, Xin Xu, Tao Zhang, Nan Bi, Luhua Wang

**Affiliations:** ^1^ Department of Radiation Oncology, National Cancer Center/National Clinical Research Center for Cancer/Cancer Hospital, Chinese Academy of Medical Sciences and Peking Union Medical College, Beijing, China; ^2^ Department of radiation Oncology, State Key Laboratory of Oncology in South China, Collaborative Innovation Center for Cancer Medicine, Sun Yat-sen University Cancer Center, Guangzhou, China; ^3^ Key Laboratory of Mass Spectrometry Imaging and Metabolomics (Minzu University of China), National Ethnic Affairs Commission, Beijing, China; ^4^ Department of radiation Oncology, National Cancer Center/National Clinical Research Center for Cancer/Cancer Hospital and Shenzhen Hospital, Chinese Academy of Medical Sciences and Peking Union Medical College, Shenzhen, China

**Keywords:** radiotherapy, immune suppression, non-small cell lung cancer (NSCLC), radio-resistance, indoleamine 2,3-dioxygenase (IDO)

## Abstract

**Objective:**

High activity of Indoleamine 2,3-dioxygenase1 (IDO1) in lung cancer patients converts tryptophan (Trp), which is the essential amino acid for T-cell metabolism, to kynurenine (Kyn) and consequently suppresses anti-tumor immune responses. We aimed to track the dynamics of IDO1 activity in stage III non-small cell lung cancer (NSCLC) patients who received first-line radiotherapy (RT) and explore its association with survival outcomes.

**Materials and methods:**

Systemic IDO1 activity was calculated by Kyn : Trp ratio. Plasma levels of Kyn and Trp in 113 thoracic RT-received stage III NSCLC patients were measured by high-performance liquid chromatography before the initiation of RT. The dynamic change of IDO1 activity was followed in 24 patients by measuring the Kyn : Trp ratio before, during, and after RT administration.

**Results:**

In 24 patients with dynamic tracking of plasma IDO1 activity, there were no significant alterations observed among the three time points (Friedman test, p = 0.13). The changing pattern of the Kyn : Trp ratio was divided into four groups: decreased consistently during RT, first increased, then decreased, increased consistently, first decreased then increased. Patients whose Kyn : Trp ratio kept decreasing or first increased then decreased were defined as the good-change group. The good-change status was identified as an independent positive factor for overall survival (OS) and progression-free survival (PFS) (p = 0.04; p = 0.01) in multivariate analysis among evaluated parameters. Patients with good change showed significantly superior local control than the bad-change group (p = 0.01, HR = 0.22). In 113 stage III NSCLC patients with pre-radiation Kyn : Trp ratio, a trend that high baseline IDO1 activity was associated with short OS was observed (p = 0.079).

**Conclusion:**

Favorable change in IDO1 activity during RT was associated with superior OS, PFS, and local control. IDO1 activity is a promising biomarker for prognosis in stage III NSCLC patients.

## Introduction

Lung cancer is the leading cause of cancer death in China, 85% of which is non-small cell lung cancer (NSCLC) (1, 2). Unresectable stage III NSCLC accounts for nearly one-third of NSCLC, and definitive chemoradiotherapy (CRT) following consolidated immunotherapy is the standard of care (SoC) for this group of patients (3). Radiotherapy (RT) not only plays a crucial role in increasing the local control of tumors by directly damaging the double strands of deoxyribonucleic acid (DNA), but also upregulates the antitumor immune response by releasing more tumor neoantigen and activating CD8^+^ T cells in the tumor microenvironment (TME) (4). Indoleamine 2, 3-dioxygenase 1(IDO1) is the key rate-limiting enzyme in Kyn pathway that converts circulating tryptophan (Trp) into kynurenine (Kyn) and its activity can be reflected by Kyn : Trp ratio in serum (5, 6). Trp is the essential amino acid for immune cell proliferation and Trp depletion results in T-cell apoptosis (7). Kyn, on the other hand, serves as a ligand of the aryl hydrocarbon receptor (AhR) and their binding can promote T regulatory cell differentiation, directly inhibiting antitumor immune responses (8). Trp downstream metabolites including Kyn, kynurenic acid and xanthurenic acid exert cytotoxic effects on CD8^+^ tumor-infiltrating lymphocytes and inhibit the proliferation of T and natural killer cells (9). Research has shown that increased IDO1 activity is detected in NSCLC patients and is associated with poorer survival (10). IDO1 activity also predicts the efficacy of chemotherapy or immunotherapy in lung cancer patients (11, 12). Kong is the first to report that reduced levels of Kyn : Trp ratios were observed during RT in stage III inoperable NSCLC patients but restored 3 months after RT completion. And the favorable change (low post/pre Kyn) and favorable baseline (low pre-RT Kyn : Trp ratio) predicted longer overall survival (OS) (13). Kong validated this result in early-stage NSCLC and pointed out that comparing with stereotactic body radiotherapy (SBRT), conventionally fractionated 3-dimensional conformal radiotherapy(3DCRT) caused more rising of absolute Kyn levels after RT, which indicated SBRT might have less immunosuppressive effect than 3DCRT (14). Another relevant study showed that pre-RT Kyn : Trp ratios and post/pre Kyn : Trp ratios were independent predictive factors for progression-free survival (PFS) and OS in all stage RT-received NSCLC patients (15). A single-arm Phase II trial involving induction chemotherapy followed by concurrent CRT in stage III NSCLC patients reported that the mean Kyn : Trp ratio increased after induction therapy or CRT and the study correlated the post-induction chemotherapy increase in IDO1 activity with worse OS (12).

Based on the aforementioned research, we assumed that RT exerts an effect on IDO1 activity. It is interesting that RT-mediated downregulation of Kyn : Trp levels is restored after RT, but the relevant mechanism is unclear yet. A study has established that the classical interferon-γ(IFN-γ) mediated Janus kinase (JAK)/signal transducer and activator of transcription 1(STAT1) signaling can be activated by RT. STAT1 has a dual function in the TME of radioresistant cells by either protecting radiation-induced damage or mediating tumor cell apoptosis, which associates STAT1 with radio-resistance (16). Intriguingly, IFN-γ signaling through JAK/STAT1 is also the primary mechanism for inducing IDO1 (17). Research reported that IDO1 is important for supporting lung cancer development, consistent with its role in inducing T cell tolerance (18). Preclinical studies demonstrated that IFN-γ resulting IDO1/aryl hydrocarbon receptor (AhR) dependent p27 induction could prevent STAT1 signaling, thus suppressing the process of tumor cell death and activating tumor dormancy program (19). Tumor cell dormancy refers to tumor cells in the equilibrium state when they remain unedited and proliferate poorly so that they can escape immune control (20). Even though the interplay between the RT-IFN-γ/JAK/STAT1 and IFN-γ/IDO1-p27/STAT1 was unclear yet, we hypothesized that high levels of IDO1 activity at baseline promote tumor outgrowth through immune escape, while the persistent high levels of IDO1 activity during RT resulted in radio-resistance by eliciting tumor-dormancy rather than STAT1-mediated tumor cell apoptosis. We explored the changing pattern of Kyn : Trp ratio and Trp pathway metabolites during RT and correlated them with OS, PFS, and local recurrence-free survival (LRFS) in RT-received unresectable stage III NSCLC patients.

## Materials and methods

### Follow-up and treatment

This study was approved by the Institutional Review Board of the National Cancer Center, Chinese Academy of Medical Sciences and Peking Union Medical College (IRB No. NCC-000302). All patients provided written informed consent before therapy. Patients who were pathologically diagnosed with unresectable stage III NSCLC as per the American Joint Committee of Cancer (AJCC) 8th edition cancer staging manual between January 2013 and December 2017 at our institution were prospectively included. All patients underwent thoracic intensity-modulated radiotherapy (IMRT). X-rays at 6 MV were used, with a median total dose of 60 Gy (28–67 Gy) in 30 (13–33) fractions. Etoposide/cisplatin and paclitaxel/carboplatin regimens were primarily used in concurrent CRT or sequential CRT. Patients were evaluated weekly during RT and followed up 1 month after the last irradiation, then every 3 months for 2 years, and then every 6 months for another 3 years. Routinely, follow-up includes blood tests, computed tomography (CT) of the chest and abdomen (enhanced requested if without contraindications), bone scans, and brain magnetic resonance imaging (MRI). The median follow-up period was 63.0 months.

### Sample and clinical data collection

The plasma of patients was collected before the initiation of RT, four weeks after RT initiation, right after the completion of RT and stored at −80 °C. At the end of the follow-up, 24 patients with dynamic monitoring at the three time points and 89 patients with pre-RT plasma were included. Clinical data, including age, gender, smoking, histology, clinical stage, Eastern Cooperative Oncology Group Performance Status Scale (ECOG-PS), biologically effective dose (BED), and treatment patterns were collected.

### Measurements of plasma Trp and Kyn

The plasma Trp and Kyn were quantified by the liquid chromatography-tandem mass spectrometry (LC-MS/MS) method. Plasma samples were collected and stored at −80 °C until analysis. Eighty microliters of plasma samples were deproteinized by 240 µl of frozen acetonitrile and 8 µl of internal standard solution, vortex mixed, and centrifuged at 10,000 rpm for 5 min. All the clean upper layers were centrifugally concentrated. Before injection into the chromatographic system, 80 µl of 2% acetonitrile solution was added to each sample, and the samples were filtered through a 96-well plate for analysis. Five microliters of clean upper layer were injected into a chromatographic system. LC-MS/MS was performed using a high-performance liquid chromatography coupled to tandem mass spectrometry (HPLC-MS/MS) system (QTRAP™ 6500, AB SCIEX, USA) equipped with a HSS T3 column (2.1 × 100 mm, 1.8 µm), with the column temperature at 35 °C. The mobile phase consisted of a solution of 0.1% aqueous formic acid (A) and 100% acetonitrile (B); elution was performed at a flow rate of 250 µl/min, using an elution gradient. The detector was set in the positive ion mode. The instrument was set in the multiple reaction monitoring (MRM) mode. Data were acquired by the Analyst 1.6.1 Software. Raw data were processed with Multiquant^®^ 2.2 software (AB SCIEX, USA) and the data was calibrated using the Norm ISWSVR program in Python 3.6.

### Primary endpoints and statistical analysis

The OS was defined as the time from the start of RT to death. PFS was defined as the time from the start of RT to the date of progression or death. LRFS was defined as the time from the start of RT to the date of local/regional recurrence or death. Patient and tumor characteristics were compared using the 𝛘^2^-test or Fisher’s exact test. The OS, PFS, and LRFS were estimated using the Kaplan–Meier method. The roles of different treatment regimens and the baseline characteristics in survival were explored using log-rank tests. A comparison of Trp metabolites between the different age groups was performed using the Mann–Whitney U test. Levels of IDO1 activity at three time points were compared using the Friedman test. The X-tile was used to find the best cutoff value (22). A Cox proportional hazards regression analysis was used to identify significant variables associated with prognosis. All P-values are two-sided, and P <0.05 was considered to indicate statistical significance. Statistical analysis was performed using IBM SPSS Statistics, version 25.0 (IBM Corp., Armonk, NY, USA) and GraphPad Prism (version 8.0.2).

## Results

### Patient characteristics

A total of 113 stage III patients with qualified plasma samples and follow-up were enrolled in the analysis. As shown in **Table 1**, the median age of the population was 62 years (range, 35–80). There were 15 (13.2%) females. Ninety-seven (85.8%) of the patients received chemotherapy during the course, and 107 (94.6%) of them received a radiation dose of no less than 50 Gy.

**Table 1:** Patient baseline characteristics.

Variables	N = 113
Sex	
male	98
female	15
Age(years)	
≥62	61
<62	52
ECOG-PS	
0–1	63
≥2	50
Smoking history	
never	21
current or former smoker	92
Clinical stage (8th)	
IIIA	26
IIIB	64
IIIC	23
PET-CT	
Yes	48
No	65
Histology	
Squamous	76
non-squamous	37
Treatment patterns	
CCRT	54
SCRT	43
RT alone	16
BED	
≥70	86
<70	27

ECOG-PS, Eastern Cooperative Oncology Group Performance Status; CCRT, concurrent chemoradiotherapy; SCRT, sequential chemoradiotherapy; RT, radiotherapy; BED, biologically effective dose.

### Pre-RT Trp pathway metabolites summary

**Table 2** details the plasma concentrations of Trp and its main downstream metabolites—Kyn between different age groups (original data about the concentrations of Kyn and Trp was provided in the **Supplementary Table 1**). The median age of the whole cohort was 62, which was used as the cutoff point to dichotomize patients. The median value of the calculated Kyn : Trp ratio was 0.07 in the whole population, and patients aged over 62 had significantly higher IDO1 activity than those aged less than 62 years old (p = 0.032). The distributions of T stage and N stage were also compared between these two groups, and no significance was observed (T stage, p = 0.375; N stage, p = 0.195).

**Table 2:** Comparisons of pre-RT serum concentrations between different age group in stage III non-small cell lung cancer patients (N = 113).

	All (N = 113)	Age ≤62 (N = 61)	Age >62 (N = 52)	p-value
Tryptophan (μmol/L)	26.70 ± 5.73a	26.53 ± 6.17	26.89 ± 5.22	0.867
Kynurenine (μmol/L)	2.05 ± 0.93	1.84 ± 0.73	2.29 ± 1.08	0.031
Kyn/Trp ratio (×100)	7.71 ± 3.18	7.07 ± 2.64	8.47 ± 3.60	0.032

a Median ± SD.

### Dynamics of IDO1 biomarkers during RT and survival outcomes

**Figure 1:**
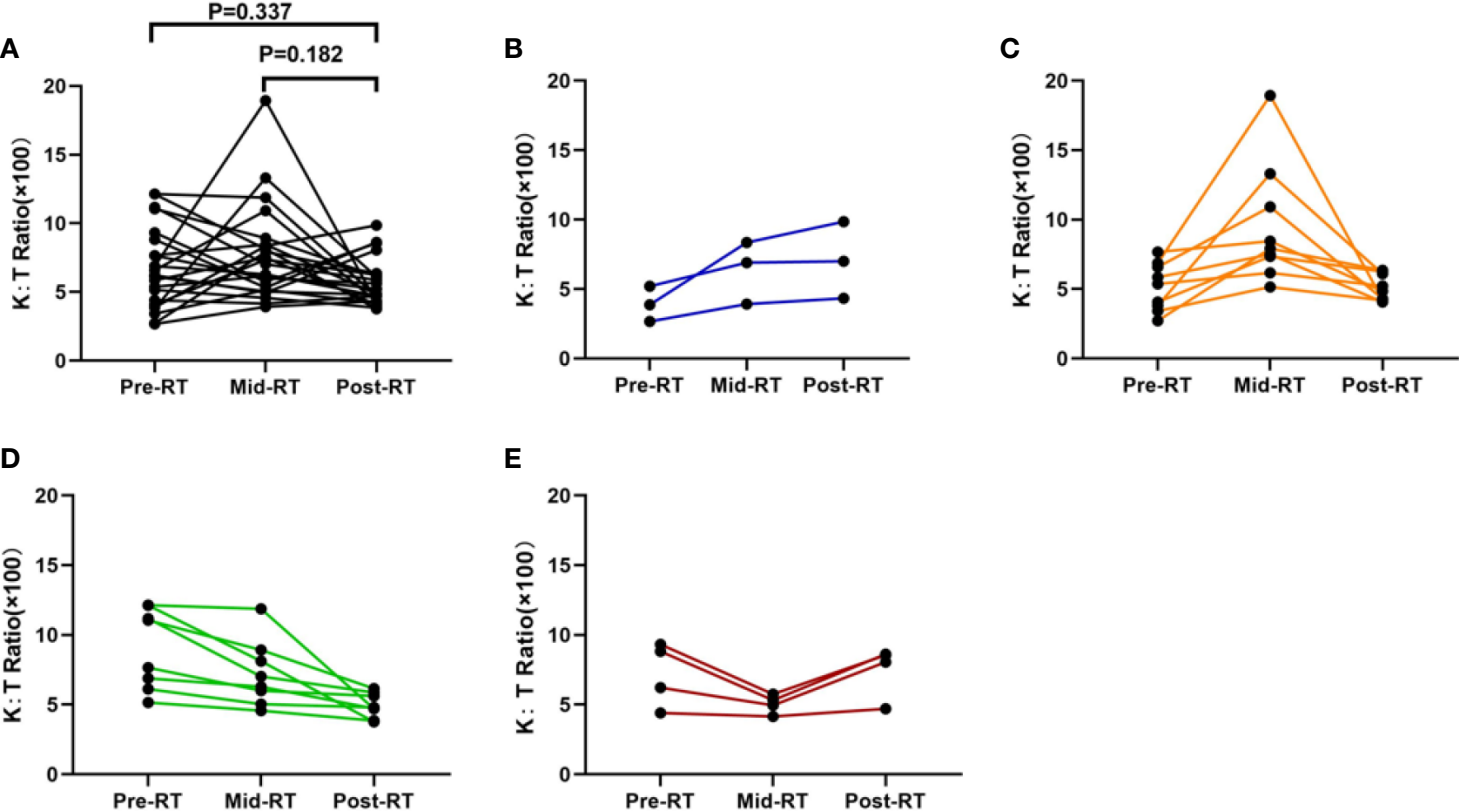
Dynamic change of IDO1 activity during radiotherapy at three time points (before the initiation of radiotherapy, two weeks after the initiation of radiotherapy and after the last irradiation). **(A)** All 24 patients with dynamic tracking during radiotherapy; **(B)** patients with constantly increasing IDO1 activity (mid-RT:pre-RT >1 and post-RT:mid-RT >1); **(C)** patients with IDO1 activity firstly increasing then decreasing (mid-RT:pre-RT >1 and post-RT:mid-RT <1); **(D)** patients with constantly decreasing IDO1 activity (mid-RT:pre-RT <1 and post-RT:mid-RT <1); and **(E)** patients with IDO1 activity firstly decreasing then increasing (mid-RT:pre-RT <1 and post-RT:mid-RT >1).

**Figure 2:**
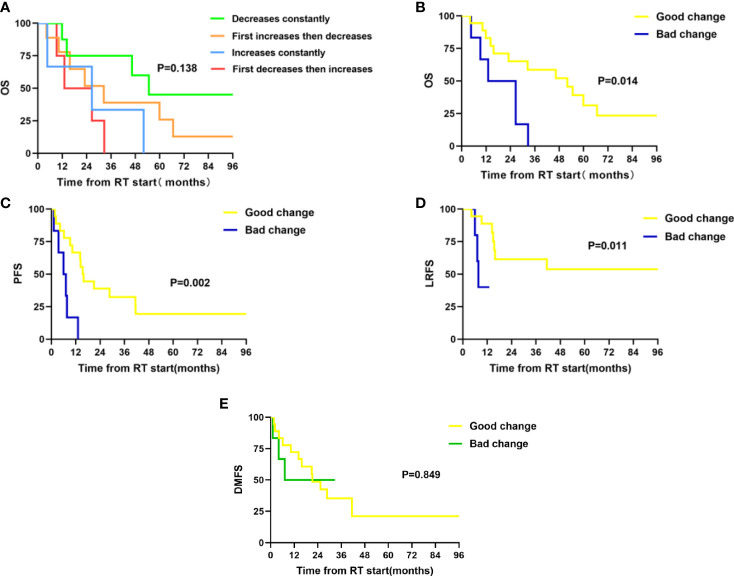
Twenty-four patients were divided into four groups according to the dynamics of Kyn : Trp ratio at three time points during radiotherapy. Patients were stratified into good-change group and bad-change group according to the survival outcomes in previously defined four groups. Good-change group includes patients with IDO1 activity decreasing constantly (mid-RT:pre-RT <1 and post-RT:mid-RT <1) or first increasing then decreasing (mid-RT:pre-RT >1 and post-RT:mid-RT <1) during RT; Bad-change group includes patients with IDO1 activity increasing constantly (mid-RT:pre-RT >1 and post-RT:mid-RT >1) or first decreasing then increasing (mid-RT:pre-RT <1 and post-RT:mid-RT >1) during RT. **(A)** Comparison of overall survival in four groups; **(B)** Comparisons of overall survival in good-change and bad-change group; **(C)** Comparisons of progression-free survival in good-change and bad-change group; **(D)** Comparisons of local recurrence-free survival in good-change and bad-change group; and **(E)** Comparisons of distant metastasis-free survival in good-change and bad-change group.

Twenty-four patients with available plasma at three time points were analyzed. Of the 24 patients, only three patients were females, and two patients never smoked. The median age was 59 years old, and 87.5% (21/24) of the patients received chemoradiotherapy, with most patients (87.5%) having a BED of no less than 70 Gy. Analysis by using the Friedman test showed that mean Kyn : Trp ratios did not change significantly during RT, as it is depicted in **Figure 1A** (p = 0.132). The levels of Trp and Kyn also remained relatively stable during RT. We then described the changing patterns of the Kyn : Trp ratio and associated them with survival outcomes. According to the dynamics of Kyn : Trp ratio at three time points, patients were divided into four groups respectively: decreased consistently during RT(mid-RT:pre-RT <1 and post-RT:mid-RT <1), first increased then decreased(mid-RT:pre-RT >1 and post-RT:mid-RT <1), increased consistently(mid-RT:pre-RT >1 and post-RT:mid-RT >1), first decreased then increased(mid-RT:pre-RT <1 and post-RT:mid-RT >1) (**Figures 1B–E**). **Figure 2A** presents survival curves of the four groups, and patients with decreasing IDO1 activity during RT yielded the best OS, while the patients with increasing activity showed worse outcomes. Based on that, patients whose Kyn : Trp ratio kept decreasing or first increased then decreased were stratified as the good-change group and the rest as the bad-change group. The median OS was 52.1 months in the good-change group and 19.8 months in the bad-change group, respectively. The good-change group correlated significantly with OS (HR = 0.32, 95% CI, 0.09–1.23; p = 0.014; **Figure 2B**), PFS (HR = 0.26, 95% CI, 0.06–1.12; p = 0.002; **Figure 2C**) and LRFS (HR = 0.22, 95% CI, 0.02–2.00; p = 0.011; **Figure 2D**). This stratification was not relevant to distant metastasis-free survival (DMFS) (p = 0.849; **Figure 2E**). The good-change group was recognized as an independent prognostic factor in the univariate and multivariate analyses for OS (p = 0.021; p = 0.036; **Table 3**) and PFS (p = 0.005; p = 0.014; **Table 4**). We also analyzed the combined predictive value of the baseline and changing pattern. Patients were divided into high-baseline groups and low-baseline groups using the median Kyn : Trp value at pre-RT as the cutoff point. Then a chi-square test was performed to evaluate the correlation between the pre-RT level of IDO1 activity and the subsequent changing pattern. The result showed that pre-RT IDO activity was not significantly different between the good-change group and the bad-change group (p = 1.000). Then patients were further divided into four groups according to high or low Kyn : Trp baseline and good or bad Kyn : Trp changing pattern. Results revealed that the p value was not significant to predict OS or PFS in univariate analysis, respectively (P = 0.246; P = 0.216). Other parameters, including gender, smoking status, or treatment regimen, did not show a prognostic value for OS or PFS.

**Table 3:** Univariate and Multivariate analyses of OS in patients with dynamic monitoring of IDO activity (n = 24).

Variable	Univariate analysis		Multivariate analysis	
	HR (95% CI)	p-value	HR (95% CI)	p-value
Gender (female vs. male)	0.19 (0.024–1.43)	0.105		
Smoking (yes vs. no)	2.40 (0.32–18.20)	0.397		
Clinical stage (IIIA vs. IIIB–IIIC)	0.46 (0.11–1.99)	0.297	0.76 (0.16–3.61)	0.725
Histology (squamous vs. non-squamous)	2.11 (0.82–5.43)	0.119	2.00 (0.76–5.26)	0.163
IDO activity change (good vs. bad)	0.27 (0.09–0.82)	0.021	0.29 (0.09–0.92)	0.036
BED (<70 vs. ≥70)	0.61 (0.17–2.14)	0.436		
Treatment (CCRT vs. SCRT or RT alone)	0.82 (0.32–2.09)	0.681		

CCRT, concurrent chemoradiotherapy; SCRT, sequential chemoradiotherapy; RT, radiotherapy; Good-change group refers to patients with IDO1 activity decreasing constantly or first increasing then decreasing during RT; Bad-change group refers to patients with IDO1 activity increasing constantly or first decreasing then increasing during RT; BED, biologically effective dose.

**Table 4:** Univariate and Multivariate analyses of PFS in patients with dynamic monitoring of IDO activity (n = 24).

Variable	Univariate analysis		Multivariate analysis	
	HR (95% CI)	p-value	HR (95% CI)	p-value
Gender (female vs. male)	0.57 (0.13-2.46)	0.448		
Smoking (yes vs. no)	1.30 (0.30-5.72)	0.725		
Clinical stage (IIIA vs. IIIB–IIIC)	0.55 (0.16–1.88)	0.337	0.90 (0.24–3.39)	0.881
Histology (squamous vs. non-squamous)	2.06 (0.82–5.17)	0.125	1.76 (0.67–4.61)	0.250
IDO activity change (good vs. bad)	0.19 (0.06–0.60)	0.005	0.22 (0.06–0.73)	0.014
BED (<70 vs. ≥70)	0.43 (0.12–1.50)	0.184		
Treatment(CCRT vs. SCRT or RT alone)	0.87 (0.36–2.13)	0.763		

CCRT, concurrent chemoradiotherapy; SCRT, sequential chemoradiotherapy; RT, radiotherapy; Good-change group refers to patients with IDO1 activity decreasing constantly or first increasing then decreasing during RT; Bad-change group refers to patients with IDO1 activity increasing constantly or first decreasing then increasing during RT; BED, biologically effective dose.

### Pre-RT IDO1 biomarkers and survival outcomes

**Figure 3:**
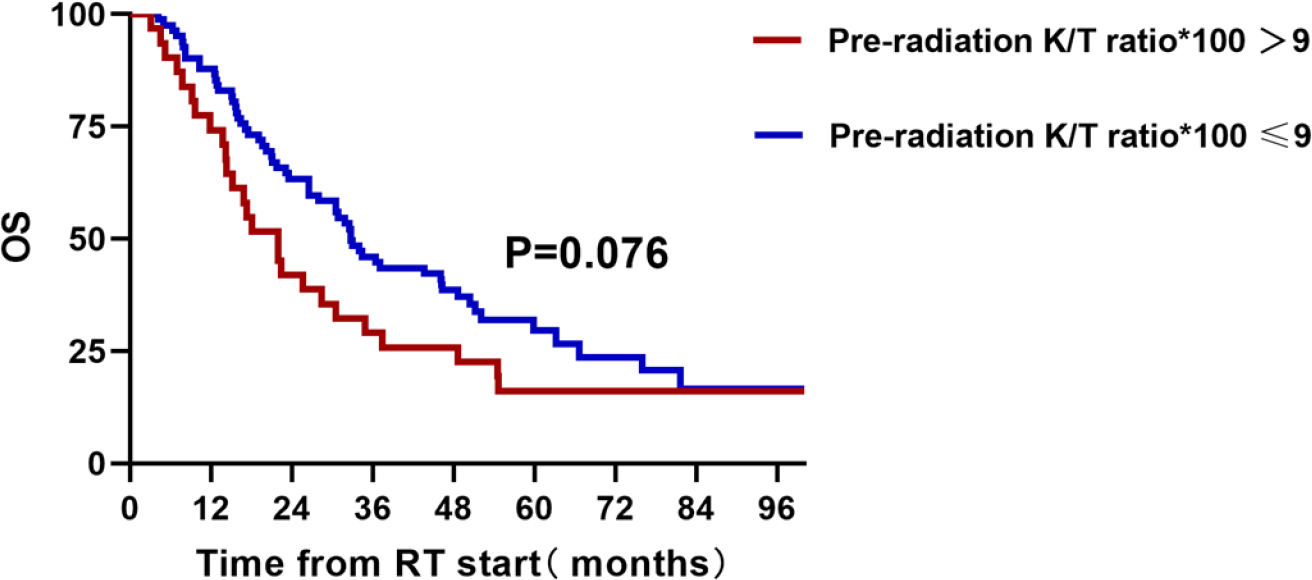
A total of 113 unresectable stage III patients with pre-radiation detection of IDO1 activity and overall survival.

The median OS and PFS of 113 patients were 30.6 (95% CI, 25.30–35.90) and 15.4 (95% CI, 12.00–18.91) months, respectively. Using X-tile method, we found that patients with a Kyn : Trp ratio less than 0.09 showed a trend for better OS (HR = 0.66, 95% CI, 0.40–1.110; p = 0.076; **Figure 3**). As shown in **Table 5**, three other parameters with a p-value less than 0.15 from univariate analysis, including gender, clinical stage, and histology, were also included in the later multivariate analysis. The p value of IDO1 activity remained non-significant in the multivariate analysis (p = 0.138). Gender difference presented a prognostic ability for OS in univariate analysis (p = 0.040) but the significance was not observed in multivariate analysis (p = 0.148).

**Table 5:** Univariate and Multivariate analyses of OS in stage III non-small cell lung cancer patients (n = 113).

Variable	Univariate analysis		Multivariate analysis	
	HR (95% CI)	p-value	HR (95% CI)	p-value
Gender (male vs. female)	2.16 (1.04–4.48)	0.040	1.76 (0.82–3.81)	0.148
Age (<62 vs. ≥62)	0.90 (0.58–1.38)	0.62		
Smoking (yes vs. no)	1.48 (0.83–2.62)	0.184		
Clinical stage (IIIA–IIIB vs. IIIC)	0.68 (0.40–1.13)	0.136	0.67 (0.40–1.13)	0.135
Histology (squamous vs. non-squamous)	1.48 (0.92–2.36)	0.103	1.34 (0.82–2.20)	0.241
Pre-radiation K/T ratio ∗ 100 (<9 vs. ≥9)	0.66 (0.42–1.05)	0.079	0.70 (0.44–1.12)	0.138
BED (<70 vs. ≥70)	1.01 (0.60–1.71)	0.959		

BED, biologically effective dose.

## Discussion

This study demonstrated that patterns of IDO1 dynamic change during RT were significantly associated with OS, PFS, and LRFS in stage III NSCLC patients. The favorable changing patterns were defined as IDO1 activity consistently decreasing or first increasing then decreasing during RT. Our study reported that the changing pattern of Kyn : Trp ratios, which represented immune status during RT, was correlated with prognosis, particularly local control, in unresectable stage III NSCLC patients from the Asian population. Relevant or similar studies are summarized in **Table 6** (5, 12–14, 23).

**Table 6:** Select studies of indoleamine 2,3 dioxygenase IDO in non-small cell lung cancer.

Year	Study	Stage	Sample size	Sample source	Dynamic tracking	Outcomes
2010	Yuzo et al.	All	N =1 23	Serum kyn/trp	No	Not significantly associated with prognosis
2013	Creelan et al.	III	N=33	Serum kyn/trp	Yes	Post-induction chemotherapy increase in K/T ratios associated with worse OS and PFS
2018	Wang et al.	III	N = 110	Serum kyn/trp	Yes	Baseline K/T ratios and changes levels of Kyn after radiotherapy significantly associated with OS
2020	Wang et al.	I–II	N = 56	Serum kyn/trp	Yes	Kyn levels after radiotherapy significantly associated with OS
2020	Zhu et al.	All	N = 104	Serum kyn/trp	Yes	Both Pre-radiation and post/pre K/T ratios associated with PFS and OS
2021	Mandarano et al.	I–III	N = 180	Serum kyn/trp	No	Not significantly associated with prognosis
2022	Our study	III	N = 113 (Baseline); N = 24 (With dynamics)	Serum kyn/trp	Yes	Of 113 patients, no significant association with OS; of 24 patients with dynamic tracking, the changing patterns of K/T ratios during radiotherapy significantly associated with OS and PFS

According to **Table 6**, despite the fact that different studies drew conflicting conclusions, most studies associated the dynamics of IDO1 activity with survival outcomes. As is illustrated in a previous study, RT can alter IDO1-mediated immune activity and the combined favorable change (low post/pre Kyn) and favorable baseline (low pre-RT Kyn : Trp ratio) were predictive of longer OS in both early stage and locally advanced NSCLC patients (13, 14). Thus, we aimed to investigate the changing pattern of Kyn : Trp ratio and Trp pathway metabolites during RT and correlate them with survival outcomes. Compared with the previous four studies, which all tracked the dynamics of IDO1 activity during RT in NSCLC patients (12–15), our study designed different monitoring time points and supported the hypothesis that changing patterns of IDO1 activity during RT were significantly associated with survival outcomes. More importantly, patients with favorable dynamics of Kyn : Trp ratio yielded significantly lower local/regional recurrence, indicating that the benefit of OS might be attributed to RT-related local control. Our finding also supported our previous hypothesis that sustainable growth of IDO1 activity during RT might be correlated with radio-resistance through IFN-γ/JAK/STAT1 pathway thus resulted in poor local control, though validated experiment required in the future. In all RT-received stage III patients, we did not find a significant correlation between pre-RT Kyn : Trp ratio levels and survival outcomes. However, a trend that higher pre-RT IDO1 activity was predictive of poorer OS was shown. This result was consistent with earlier reports (5, 12). Moreover, our data found that patients aged over the median age of the whole cohort had significantly higher Kyn : Trp ratios and Kyn levels. This was in accordance with previous research illustrating that the Kyn : Trp ratio was higher in over 68-year-old patients (68 as the median age of the study population) (5). Older people have a higher Kyn : Trp ratio and Kyn in plasma can be explained by immunosenescence, which refers to a process where functional competence of the immune system gradually declines with aging (24). Some studies documented that Kyn : Trp ratio was considered as a biomarker for aging and age-related tissue damage can lead to inflammation, acting as a stimulus for Kyn pathway (25, 26).

The PACIFIC study shifted the SoC for stage III NSCLC patients and the survival benefit with consolidation durvalumab is probably related to the control of extra-thoracic diseases (occult micro-metastases) (27). Despite advances in reducing the risk of distant metastasis, several historical attempts have failed to improve local control and survival. NRG/RTOG0617 trial tried to optimize local control by increasing the RT dose to 74 Gy, beyond the standard 60–66 Gy once-daily fraction RT. Nevertheless, the results showed that the high-dose group was not better than the standard-dose group and even led to a higher incidence of treatment-related grade ≥3 dysphagia and esophagitis (28, 29). A secondary study of RTOG0617 demonstrated that higher radiation doses to the immune cells correlated with worse OS and LPFS due to radiation-induced immunosuppression (30), which complied with previous studies showing that IDO1 activity remained at low levels in the middle phase of RT but increased after higher doses (13). Other attempts, including post-CCRT surgical resection or alteration of the RT scheme from once daily to twice daily, failed because PFS only improved marginally at the expense of quality of life (31). Our research showed that the dynamics of IDO1 activity during RT predicted LRFS and was related to radio-resistance, which shed light on the current dilemma and inspired a new strategy for combination therapy. On one hand, IDO1 activity in plasma was easily available with minor harm to the human body, so it could serve as a convenient biomarker for prognosis and efficacy. On the other hand, considering increasing levels of Kyn : Trp ratio, correlated with shorter LRFS, combining IDO1 inhibitors with radiotherapy and immunotherapy might be a promising tactic to decrease the risk of local recurrence in the future. Preclinical studies suggested that the addition of IDO1 blockade into local RT reduced tumor growth, decreased regulatory T cells, and even reversed T-cell exhaustion in the TME with good tolerance (32, 33). The experiment in the Lewis lung cancer mouse model also revealed that a double treatment regimen consisting of IDO1 inhibitor 1−methyl−tryptophan (1MT) and 10 Gy RT generated a synergistic effect and was more effective than either treatment alone (33). A phase I trial, which combined IDO1 inhibitor indoximod either with temozolomide or with radiation in children with progressive malignant brain tumors, reported that this combination was well tolerated with good quality of life (34). The study by Wang mentioned that differences in RT techniques or factions also play a role in shaping IDO1 activity and immune status (14). A higher kyn/trp ratio could predict resistance to anti-PD-1 treatment in NSCLC patients (11, 35). Clinical trials are ongoing to explore the possibility of concurrent immunotherapy and radiotherapy (KEYLYNK-012). Therefore, a large-scale prospective study is needed to find the possibility and optimal scheme for triple-regimen therapy. It is worth noting that the high expression of indoleamine 2,3-dioxygenase 2 (IDO2), an analog of IDO1, has been reported to significantly correlate with high PD-L1 levels and poor prognosis in NSCLC patients, indicating it will be another innovative target in the future (36, 37).

Our study has some limitations. Firstly, this study was conducted in a single center with a small sample size, indicating that the power of statistical analysis was not ideally sufficient with inherently introduced bias. Secondly, the starting time point for plasma collection was designed to be pre-RT, so the baseline levels of IDO1 activity in patients who had undergone induction chemotherapy were unknown and not considered. Besides, not all patients included in our study had definitive thoracic radiotherapy, though 94.6% (107/113) of the whole cohort had received an RT dose of no less than 50 Gy. Last but not least, using the Kyn : Trp ratio to measure IDO1 activity was only an indirect way since there are two other known enzymes, IDO2 and tryptophan-2,3-dioxygenase (TDO), that can catalyze the same Trp metabolic reaction. In particular, IDO2 activity status has been genetically linked to radio-responsiveness in pancreatic cancer patients (38). Although IDO1 was reported to be the most potent enzyme in the reaction (39), the interpretation of data in our study could be confounded by these other factors.

To conclude, this study demonstrated that favorable dynamics of IDO1 activity during RT were associated with superior OS, PFS, and local control. IDO1 activity is a promising biomarker for prognosis, and IDO1 inhibitors could play a role in multidisciplinary therapy in the future.

## Data availability statement

The data presented in the study are deposited in the Metabolights repository, accession number(MTBLS5267).

## Ethics statement

The studies involving human participants were reviewed and approved by the National Cancer Center, Chinese Academy of Medical Sciences and Peking Union Medical College. The patients/participants provided their written informed consent to participate in this study.

## Author contributions

LiW and LuW contributed to conception and design of the study. TZ and XX organized the database. NB performed the statistical analysis. LiW wrote the first draft of the manuscript. YC, MQ, LiW, and NB wrote sections of the manuscript. All authors contributed to manuscript revision, read, and approved the submitted version.

## Funding

This work was supported by the National Natural Sciences Foundation Key Program (81872474); the Capital’s Funds for Health Improvement and Research (2020-2-4022); and the Sanming Project of Medicine in Shenzhen (No. SZSM201612063).

## Conflict of interest

The authors declare that the research was conducted in the absence of any commercial or financial relationships that could be construed as a potential conflict of interest.

## Publisher’s note

All claims expressed in this article are solely those of the authors and do not necessarily represent those of their affiliated organizations, or those of the publisher, the editors and the reviewers. Any product that may be evaluated in this article, or claim that may be made by its manufacturer, is not guaranteed or endorsed by the publisher.
