# Supplementary material for: Dynamic change of IDO1 activity predicts survival in patients with unresectable stage III NSCLC and chemoradiotherapy
Source: Front Immunol. 2022 Aug 10;13:906815. doi: 10.3389/fimmu.2022.906815 (PMC9399602; doi:10.3389/fimmu.2022.906815)
Supplement: Supplementary file 1 [file Table_1.docx]

| **Supplementary Table 1. The concentrations of Tryptophan and Kynurenine in 113 patients** | |
| --- | --- |
| **Tryptophan(umol/L)** | **Kynurenine(umol/L)** |
| 22.9905 | 4.5577 |
| 27.4049 | 4.4025 |
| 26.9004 | 4.2373 |
| 21.7116 | 3.3821 |
| 33.29 | 5.0347 |
| 29.7774 | 4.1879 |
| 26.6807 | 3.6521 |
| 16.7935 | 2.2984 |
| 29.4352 | 3.7363 |
| 24.239 | 3.0351 |
| 21.3804 | 2.6481 |
| 30.4016 | 3.6911 |
| 30.9089 | 3.75 |
| 31.1523 | 3.5274 |
| 26.6835 | 2.9863 |
| 25.5445 | 2.857 |
| 11.4364 | 1.2784 |
| 32.2149 | 3.5779 |
| 20.6597 | 2.2847 |
| 25.6327 | 2.7896 |
| 37.6812 | 4.01 |
| 31.1621 | 3.2802 |
| 29.1817 | 2.8978 |
| 32.5001 | 3.2253 |
| 25.9785 | 2.5495 |
| 31.5355 | 3.079 |
| 27.4319 | 2.6484 |
| 12.8991 | 1.2401 |
| 21.6591 | 2.0533 |
| 17.1782 | 1.6002 |
| 28.9428 | 2.6321 |
| 26.6978 | 2.368 |
| 12.19 | 1.0755 |
| 27.2196 | 2.3417 |
| 21.4557 | 1.7914 |
| 30.5727 | 2.526 |
| 33.6681 | 2.7681 |
| 21.8142 | 1.785 |
| 35.3278 | 2.8444 |
| 26.4659 | 2.0858 |
| 21.5784 | 1.6783 |
| 32.3784 | 2.4898 |
| 26.5329 | 2.036 |
| 19.8887 | 1.5211 |
| 29.5295 | 2.2503 |
| 21.6473 | 1.6219 |
| 28.6461 | 2.1232 |
| 23.2657 | 1.7144 |
| 24.0135 | 1.7642 |
| 22.1581 | 1.6256 |
| 33.2694 | 2.4356 |
| 25.0318 | 1.8186 |
| 32.763 | 2.3253 |
| 30.5598 | 2.1293 |
| 18.5477 | 1.2777 |
| 26.4337 | 1.8168 |
| 22.6625 | 1.5569 |
| 18.0685 | 1.2389 |
| 32.0889 | 2.1979 |
| 26.9861 | 1.826 |
| 19.3561 | 1.2895 |
| 29.1185 | 1.9223 |
| 28.663 | 1.8922 |
| 37.7711 | 2.4872 |
| 24.1761 | 1.5886 |
| 27.3667 | 1.7943 |
| 21.217 | 1.3758 |
| 33.8023 | 2.1594 |
| 34.8092 | 2.1882 |
| 24.0661 | 1.5095 |
| 26.465 | 1.6498 |
| 33.2987 | 2.0698 |
| 35.954 | 2.2126 |
| 32.0174 | 1.9638 |
| 26.4129 | 1.6155 |
| 22.6594 | 1.3659 |
| 36.6271 | 2.1721 |
| 28.1727 | 1.6678 |
| 34.0824 | 2.0098 |
| 20.3466 | 1.1973 |
| 18.9932 | 1.1116 |
| 27.3289 | 1.5991 |
| 26.134 | 1.4828 |
| 28.509 | 1.6072 |
| 31.4537 | 1.7647 |
| 18.9656 | 1.0518 |
| 27.85 | 1.5122 |
| 40.0253 | 2.1678 |
| 29.5297 | 1.5889 |
| 29.3439 | 1.5757 |
| 21.9467 | 1.1742 |
| 19.8236 | 1.0227 |
| 18.2513 | 0.9407 |
| 24.1073 | 1.2246 |
| 22.9668 | 1.147 |
| 28.4283 | 1.418 |
| 25.1204 | 1.2305 |
| 35.2443 | 1.7231 |
| 25.7861 | 1.2589 |
| 28.1088 | 1.3288 |
| 33.0654 | 1.4722 |
| 15.6784 | 0.6908 |
| 35.0258 | 1.5371 |
| 33.5869 | 1.4303 |
| 30.7091 | 1.3047 |
| 25.9406 | 1.0578 |
| 24.3967 | 0.9433 |
| 32.3663 | 1.2345 |
| 20.6795 | 0.7878 |
| 23.0806 | 0.7888 |
| 24.6781 | 0.6723 |
| 28.5456 | 0.7628 |
| 21.7591 | 0.5623 |
